# BMC Nursing reviewer acknowledgement, 2012

**DOI:** 10.1186/1472-6955-12-4

**Published:** 2013-03-11

**Authors:** Thomas A Rowles

**Affiliations:** 1 BioMed Central, Floor 6, 236 Gray's Inn Road, London WC1X 8HB, United Kingdom

## Abstract

**Contributing reviewers:**

The editors of BMC Nursing would like to thank all our reviewers who have contributed to the journal in Volume 11 (2012).

## 

Jennifer Abbey

Australia

Gerd Ahlstrom

Sweden

Adenike Akhigbe

Nigeria

Noori Akhtar-Danesh

Canada

Sharon Andrew

United Kingdom

Monireh Anoosheh

Iran

Alan Astrow

USA

Megan Bair-Merritt

USA

Andrea Baumann

Canada

Paul Bennett

Australia

Carina Berterö

Sweden

Kristín Björnsdóttir

Iceland

Carol Bond

United Kingdom

Petra Brysiewicz

South Africa

Dympna Casey

Ireland

Katia Castetbon

France

Sally Chan

Singapore

Jih Chen

Taiwan

Collette Clifford

United Kingdom

Simon Cooper

Australia

Patricia Davidson

Australia

Abbas Ebadi

Iran

Karen-Leigh Edward

Australia

Helen Edwards

Australia

Sherry Espin

Canada

Jason Etchegaray

USA

Mohamad Fakih

USA

Kathleen Finlayson

Australia

Jan Florin

Sweden

Karen Francis

Australia

Brigid Gillespie

Australia

Laurie Grealish

Australia

Beverly Green

USA

Trisha Greenhalgh

United Kingdom

Elizabeth Halcomb

Australia

Ruud Halfens

Netherlands

Virpi Susanna Hantikainen

Switzerland

Jun Hasegawa

Japan

Ciara Hughes

United Kingdom

Ewa Idvall

Sweden

Maree Johnson

Australia

Annikki Jonsson

Sweden

Lynn Kemp

Australia

Alison Kitson

Australia

Doris Leung

Hong Kong

Kim Lützén

Sweden

Tanja Manser

Switzerland

Lih-Wen Mau

USA

Katherine Mcgilton

Canada

David Mellor

Australia

Mark Meterko

USA

Jane Mills

Australia

Mark Mitchell

United Kingdom

James Naessens

USA

Reza Negarandeh

Iran

Per Nilsen

Sweden

Linda Ogilvie

Canada

Shaun O'Keeffe

Ireland

Linda O'Mara

Canada

Margareta Östman

Sweden

Dula Pacquiao

USA

Alvisa Palese

Italy

John Paley

United Kingdom

Mary Palmer

USA

Guadalupe Palos

USA

Evridiki Papastavrou

Cyprus

Ruth Paris

USA

Rhian Parker

Australia

Matthew Parsons

New Zealand

Christopher Pearce

Australia

Lin Perry

Australia

Barbara Pesut

Canada

Daniel Pesut

USA

Anastas Philalithis

Greece

Barbara Pieper

USA

Vasilios Raftopoulos

Cyprus

Lois Ramondetta

USA

Virginia Rice

USA

Carolyn Ross

Canada

Trudy Rudge

Australia

Kathy Rush

Canada

Mahvash Salsali

Iran

Stefan Sävenstedt

Sweden

Virginia Schmied

Australia

Shuh-Jen Sheu

Taiwan

Dong Wook Shin

South Korea

Souraya Sidani

Canada

Olle Söderhamn

Norway

Stephanie Sogg

USA

Allison Squires

USA

Jayadevan Sreedharan

United Arab Emirates

Victoria Stanhope

USA

Melissa Thong

Netherlands

Annie Topping

United Kingdom

Athanasios Tselebis

Greece

Flora Tzelepis

Australia

Kim Jocelyn Usher

Australia

Mojtaba Vaismoradi

Iran

Roger Watson

United Kingdom

Douglas Wiebe

USA

Clare Wilding

Australia

## Pre-publication history

The pre-publication history for this paper can be accessed here:

http://www.biomedcentral.com/1472-6955/12/4/prepub

